# Enhancement of Toxic Efficacy of Alkylated Polycyclic Aromatic Hydrocarbons Transformed by *Sphingobium quisquiliarum*

**DOI:** 10.3390/ijerph17176416

**Published:** 2020-09-03

**Authors:** So-Young Lee, Jung-Hwan Kwon

**Affiliations:** Division of Environmental Science and Ecological Engineering, Korea University, 145 Anam-ro, Seongbuk-gu, Seoul 02841, Korea; smithy1028@hanmail.net

**Keywords:** polycyclic aromatic hydrocarbons (PAHs), biodegradation, biotransformation, aquatic toxicology, oil spills

## Abstract

Alkylated polycyclic aromatic hydrocarbons (PAHs) are abundant in crude oils and refined petroleum products and are considered as major contributors to the toxicity of spilled oils. In this study, the microbial degradation of model (alkylated) PAHs (i.e., phenanthrene, 3-methylphenanthrene, 3,6-dimethylphenanthrene (36DMPhe), pyrene, and 1-methylpyrene (1MP)) by the bacterium *Sphingobium quisquiliarum* EPA505, a known degrader of PAHs, was studied. To evaluate the toxic potential of the metabolic products, reaction mixtures containing metabolites of 36DMPhe and 1MP were fractionated by high-performance liquid chromatography, and their effects on the luminescence inhibition of *Aliivibrio fischeri* were evaluated. Although the luminescence inhibition of 36DMPhe and 1MP at their solubility levels was not observed, inhibition was observed in their metabolite fractions at the solubility limit of their parent molecule. This indicates that initial biotransformation increases the toxicity of alkylated PAHs because of the increased solubility and/or inherent toxicity of metabolites. Qualitative analysis of the metabolite fractions suggested that mono-oxidation of the methyl group is the main metabolic pathway of 36DMPhe and 1MP.

## 1. Introduction

Polycyclic aromatic hydrocarbons (PAHs) are ubiquitous environmental toxicants with various emission sources [1,2,3]. Alkylated PAHs are more abundant than their unsubstituted forms in crude oil and refined petroleum products and are thought to be major contributors to the overall toxicity of spilled oil [4,5]. However, the environmental fate and transformation of alkylated PAHs after spills or other contamination requires further elucidation.

In the environment, oils undergo various weathering processes (e.g., spreading, dispersion, dissolution, evaporation, emulsification, sinking, biodegradation, and photodegradation), resulting in changes of chemical composition. Biodegradation plays a very important role in the complete mineralization of hydrocarbons. According to calculations of the remaining oil in the Gulf of Mexico after the Deepwater Horizon oil spill, for example, it was estimated that hydrocarbon-degrading bacteria removed up to approximately 50% of the hydrocarbons [6,7,8].

It is well-known that many aerobic bacteria degrade aromatic hydrocarbons as substrates [9]. Many bacterial strains have been evaluated for their ability to degrade aromatic hydrocarbons including PAHs via one of the three methods: mineralization, co-metabolic transformation, and non-specific oxidation [10]. Among these, *Sphingomonas* sp. strains can degrade PAHs with 2–5 rings, which are major PAHs of toxicological concerns after spills, via non-specific oxidation utilizing enzymes such as dioxygenase or other oxygenase [11]. Although the biodegradation of unsubstituted PAHs by microorganisms has been studied for decades [9,12,13,14,15], aerobic biodegradation of alkylated derivatives has not been widely examined and studies have mainly focused on methylated naphthalenes [16,17,18,19,20,21,22]. Because alkylated phenanthrenes and pyrenes are important toxicants in oil contamination, studies are needed to identify their biodegradation and transformation products during weathering processes. In a few studies, the biodegradation kinetics of alkylated PAHs were investigated together with their unsubstituted forms [23,24,25,26,27]. Zhong et al. showed that microbial transformation of 1-methylphenanthrene by *Sphingobium* sp. MP9-4 occurs by the simultaneous monooxygenation of the methyl group and dioxygenation on unsubstituted benzene ring [22]. However, metabolic transformation studies have been conducted for only a few alkylated PAHs, whereas there are many other alkylated PAHs present in spilled oils [28,29,30]. Further studies are needed to understand whether oxidation pathways in aerobic bacteria such as *Sphingobium* sp. can be generalized to various alkylated PAHs.

It is generally accepted that oxygenated PAHs (oxy-PAHs) are generated by degradation of PAHs in aerobic environments and that they are more polar than unsubstituted PAHs. Oxy-PAHs have a higher water solubility and thereby higher bioavailability compared to unsubstituted PAHs [31,32]. Although alkylated PAHs have very low water solubility, the transformation products are expected to be more soluble, resulting in increased exposure of organisms if water solubility limits chemical exposure. Moreover, the carcinogenicity of PAHs is mediated by their reactive transformation products. As concerns regarding environmental effects have increased, the ecotoxicity of oxy-PAHs including hydroxylated PAHs, aromatic acids, and ketone- and Quinone-substituted PAHs has been investigated [33,34,35,36,37,38,39]. Fallahtafti et al. examined the chronic toxicity of 1-methylphenanthrene and its hydroxylated derivatives to Japanese medaka (*Oryzias latipes*), showing that ring hydroxylation enhanced the toxicity of PAHs [35]. Similar results were observed in developmental toxicity tests of zebrafish embryo by chrysene and its hydroxylated derivatives, revealing that hydroxylated PAHs are more toxic than unsubstituted PAHs [37].

The main goals of this study were to (1) determine and compare the biodegradation kinetics of alkylated phenanthrenes and pyrenes with those of unsubstituted forms, (2) identify major biodegradation metabolites, and (3) evaluate the toxic potency and efficacy of identified metabolites. Two alkylated phenanthrenes, 3-methylphenanthrene (3MPhe) and 3,6-dimethylphenanthrene (36DMPhe), and 1-methylpyrene (1MP) were chosen as model alkylated PAHs and the bacterium *Sphingobium quisquiliarum* EPA505 was used to degrade PAHs. The biodegradation kinetics of alkylated PAHs and their parent PAHs was assessed in batch experiments. The main metabolites of selected alkylated PAHs were enriched by high-performance liquid chromatography (HPLC) fractionation and identified by gas chromatography-mass spectrometry (GC-MS). The toxicity of metabolic fractions was also measured by determining the luminescence inhibition of *Aliivibrio fischeri* as an ecotoxicity endpoint and compared to that of parent compounds.

## 2. Materials and Methods

### 2.1. Materials

GC-grade 3-methylphenanthrene (98%), 3,6-dimethylphenanthrene (95%), and 1-methylpyrene (94%) were purchased from Tokyo Chemical Industry Co. (Tokyo, Japan). Analytical-grade phenanthrene (97%), pyrene (99%), fluoranthene (98%), dimethyl sulfoxide (DMSO; 99.5%) and a solution of *N*-*tert*-butyldimethylsilyl-*N*-methyltrifluoroacetamide with 1% *tert*-butyldimethylchlorosilane (MTBSTFA, ≥95%) were purchased from Sigma-Aldrich (St. Louis, MO, USA). Bioreagent-grade nitrilotriacetic acid (≥99.0%), potassium phosphate dibasic (≥99.0%), sodium phosphate dibasic (≥99.0%), calcium chloride dihydrate (≥99.0%), iron(ΙΙ) sulfate heptahydrate (≥99.0%), magnesium sulfate heptahydrate (≥99.0%), ammonium molybdate tetrahydrate (≥99.0%), zinc sulfate heptahydrate (≥99.0%), copper(ΙΙ) chloride dihydrate (≥99.0%), Ethylenediaminetetraacetic acid disodium salt dihydrate (98.5–101.5%), cobalt(ΙΙ) chloride hexahydrate (98–102%), sodium borohydride (99%), manganese(ΙΙ) sulfate hydrate (≥99.0%) and ammonium sulfate (≥99.0%) were purchased from Sigma-Aldrich (St. Louis, MO, USA). The medical-grade polydimethylsiloxane (PDMS) sheet (1 mm thickness, density of 1170 kg m^−3^) was purchased from Specialty Silicone Products, Inc. (Ballston Spa, NY, USA). Methanol (HPLC-grade) was purchased from Honeywell Burdick & Jackson (Ulsan, Korea). Acetonitrile (HPLC ultra-gradient solvent) was purchased from Avantor Performance Materials, Inc. (Center Valley, PA, USA).

The minimal salts medium was prepared by adding following three solutions per liter: (1) 40 mL of buffer solution containing 134 g of Na_2_HPO_4_ and 68 g of KH_2_PO_4_ per liter (pH adjusted to 7.25 using KOH), (2) 10 mL of mineral base containing 10 g of nitrilotriacetic acid, 14.45 g of MgSO_4_∙7H_2_O, 3.33 g of CaCl_2_∙2H_2_O, 0.00925 g of (NH_4_)_6_Mo_7_O_24_∙4H_2_O, 0.099 g of FeSO_4_∙7H_2_O, and 50 mL of Metal “44” solution per liter (pH adjusted to 7.25 using KOH), and (3) 10 mL of 100 g L^−1^ of (NH_4_)_2_SO_4_ solution [40,41].

### 2.2. Culturing of Bacteria

*Sphingobium quisquiliarum* DSM 7526 (strain EPA505) was selected as the test species for biodegradation tests and was purchased from DSMZ (Braunschweig, Germany). A custom-cut PDMS sheet (5 × 5 cm) was used to supply fluoranthene to the bacterial growth medium by passive dosing [42,43,44]. To saturate the PDMS with fluoranthene, PDMS sheets were submerged in methanol solution containing excess crystals of fluoranthene in a glass bottle. The bottle was placed on a horizontal shaker at 150 rpm and 25 °C for 24 h. After shaking, the PDMS sheets were removed from the methanol solution and air-dried in a clean bench. A frozen supply of *S. quisquiliarum* was plated onto LB agar plates and incubated at 28 °C. After incubation, a single colony was picked and inoculated into a glass bottle containing 50 mL of minimal salt medium and a fluoranthene-loaded PDMS sheet. The bottle was closed loosely with a cap and incubated at 25 °C and 120 rpm for 4 days. After incubation, 0.8-mL aliquots of medium containing the grown biomass were dispended into a sterile external cryovial (Thermo Fischer Scientific, Waltham, MA, USA). The medium was then mixed with 0.8 mL of glycerol and stored at −80 °C until use.

### 2.3. Biodegradation Kinetics

Thirty milliliters of minimal salts medium were added to a glass bottle containing fluoranthene-saturated PDMS and inoculated with 200 μL of a thawed aliquot of strain EPA505. The bottle was loosely closed with a cap and placed on a horizontal shaker at 25 °C and 120 rpm. After 4 days, the grown biomass suspension was diluted to 1/100 with minimal salts medium. This suspension was also serially diluted, plated on LB agar plates, and incubated for 3 days for colony counting.

For biodegradation tests, an individual compound dissolved in DMSO was spiked into 1 L of the strain EPA 505 culture (2.54 × 10^7^ and 5.78 × 10^7^ cfu mL^−1^ for phenanthrenes and pyrenes, respectively) at the initial concentration close to its aqueous solubility limit (Table 1). The DMSO content in the medium was 0.1% by volume. After mixing the spiked medium, 5 mL of the sample was collected and added to a 20 mL glass vial with a Teflon-coated screw cap. The biodegradation test was initiated by placing the test vials on a rotary shaker (200 rpm) at 25 °C in the dark. All samples were prepared in triplicate, and the negative control prepared with sterilized cells was included in all tests. Three vials were used to detect the concentrations of remaining compounds at 0, 1.5, 3, 4.5, and 6 h after initiating the biodegradation test for phenanthrene, 3-methylphenanthrene, and 3,6-dimethylphenanthrene, and at 0, 9, 12, 15, 18, and 24 h after initiation for pyrene and 1-methylpyrene. Bacterial biomass at the end of the tests was counted to assure that bacterial activity was not altered during the tests. At each sampling time, the whole medium was extracted using 3 × 20 mL hexane:ethylacetate (7:3, *v*/*v*). The extract was then passed through a sodium sulfate layer and regenerated cellulose membrane filter (pore size 0.2 µm, diameter 47 mm, chmlab group, Barcelona, Spain). The filtered extract was evaporated using a rotary evaporator to approximately 2 mL and the remaining solution was evaporated under a gentle nitrogen gas stream to complete dryness. The dried residue was re-dissolved in 1 mL methanol for ultra-performance liquid chromatography (UPLC) analysis.

The concentrations of PAHs and alkylated PAHs was quantified using an Acquity UPLC^®^ (Waters, Milford, MA, USA) equipped with a Sample manager-FTN, Quaternary Solvent Manager, and FLR Detector. The mobile phase in isocratic mode was 70% acetonitrile and 30% water at a flow rate of 0.2 mL min^−1^ at ambient temperature. The target compounds were separated on an Acquity UPLC^®^ BEH C18 column (2.1 × 50 mm, 1.7 µm particle size; Waters) at 40 °C. PAHs and alkylated PAHs were detected using the fluorescence detector with excitation (λ_ex_) and emission wavelengths (λ_em_) of 260 and 352 nm for phenanthrene, 3MPhe, and 3,6DMPhe and 260 and 420 nm for pyrene and 1MP.

In quantitative analysis of target compounds, the standard and blank samples were analyzed every 15 samples for quality control. No target peaks were observed in all blank samples and the relative standard deviations of peak areas for chemical standards during the analysis were 4.42%, 4.18%, 6.33%, 3.11% and 4.47% for phenanthrene, 3MPhe, 3,6DMPhe, pyrene, and 1MP, respectively.

The extraction recoveries at solubility level were 82.0 ± 1.4%, 90.3 ± 3.5%, 83.7 ± 1.5%, 94.3 ± 2.5%, and 88.3 ± 1.9% for phenanthrene, 3MPhe, 3,6DMPhe, pyrene, and 1MP, respectively.

### 2.4. Fractionation of Transformation Products

Following the biodegradation kinetic tests, the metabolic products of 3,6-dimethylphenanthrene and 1-methylpyrene were qualitatively identified in the UPLC chromatograms. To evaluate the toxicity of metabolic products based on the luminescence inhibition of *A. fischeri*, the column effluent containing metabolites was enriched. A 1-L bottle containing 800 mL medium and *S. quisquiliarum* spiked with 3,6-dimethylphenanthrene or 1-methylpyrene at their aqueous solubility levels (Table 2) was placed on a rotary shaker (200 rpm) at 25 °C for at least for 24 h. After incubation, the solution was passed through an Oasis^®^ HLB (200 mg) cartridge (Waters). The loaded cartridge was eluted with 4 mL methanol for metabolites from 3,6-dimethylphenanthrene or 4 mL dichloromethane for metabolites from 1-methylpyrene. The eluent was filtered through a 0.2-µm regenerated cellulose membrane filter (chmlab group), and then re-dissolved in 4 mL methanol after evaporation of the extraction solvent using a rotary evaporator and nitrogen gas stream for 3D-scanning (fixed λ_ex_ at 260 nm) with a fluorescence detector (FLD). Based on the peaks identified by 3D-scanning, the eluent was further fractionated using a Waters HPLC system equipped with a Waters 717+ autosampler, Waters 2475 multi λ fluorescence detector, and two Waters 515 HPLC pumps. Fifty microliters of eluent were injected into the HPLC system, and the metabolites were separated on a Thermo C18 column (4.6 × 150 mm, 5 μm particle size; Thermo Fisher Scientific). The mobile phase in isocratic mode was 90% acetonitrile and 10% water (*v/v*) at a flow rate of 1 mL min^−1^ at ambient temperature.

The effluent fraction containing metabolite peaks under FLD was collected. The fractionation procedure was repeated 40 times to obtain a sufficient mass of metabolites for chemical identification. The combined column effluent was passed through a sodium sulfate layer, followed by evaporation of the solvent using a rotary evaporator and a gentle nitrogen stream, and finally re-dissolved in 2 mL ethyl acetate for GC-MS analysis.

### 2.5. Identification of Metabolic Products of Alkylated PAHs

To identify metabolites of 3,6-dimethylphenanthrene and 1-methylpyrene, the samples were derivatized with MTBSTFA, which is frequently used to detect phenolic metabolites. One milliliter of ethyl acetate solution containing the metabolites was collected into a 2-mL vial. After evaporation of the ethyl acetate under a gentle nitrogen stream, the residue was reacted by spiking 20 µL MTBSTFA solution in a water bath at 60 °C for 60 min. After the reaction was complete, 480 μL ethyl acetate was added for GC-MS identification. Metabolites of alkylated PAHs were identified using an Agilent Technologies model 7890 gas chromatograph (GC) with a HP-5MS capillary column (30 m, 0.25 mm, 0.25 µm; Agilent Technologies), coupled with a model 5975 mass spectrometer (MS). The GC column temperature was held at 100 °C (1 min), increased to 160 °C at a rate of 15 °C min^−1^, increased to 300 °C at a rate of 4 °C min^−1^, and then held for 2 min. The inlet was held at 280 °C and used in splitless mode. Helium was used as a carrier gas at a rate of 1 mL min^−1^. The GC-MS interface temperature was maintained at 280 °C. The MS was used in electron impact mode (70 eV), and scans ranged from 50 to 500 *m/z*. The ion source and mass filter temperatures were held at 230 °C and 150 °C, respectively. Alkylated PAHs metabolites were identified by comparison with authentic and/or general standards and the literature. The metabolites identified in the fractionated samples were not detected in controls.

### 2.6. Luminescence Inhibition Assay

A Microtox^®^ Model 500 system (Strategic Diagnostics, Inc., Newark, DE, USA) was used to measure the luminescence inhibition of *A. fischeri* by detecting the metabolic products of 3,6-dimethylphenanthrene and 1-methylpyrene. Freeze-dried *A. fischeri* and saline diluent (2% NaCl) were purchased from Strategic Diagnostics, Inc. Two hundred microliters of ethyl acetate concentrate were added to a 4-mL glass vial, and the solvent was evaporated under a gentle nitrogen stream. The dried residue was dissolved in 2 mL of HPLC-grade water and this dissolved mixture solution was used for the Microtox^®^ assay without further treatment. Luminescence inhibition after 15 min of exposure was measured according to the manufacturer’s protocol. Dose-response curves were derived using Microtox^®^ Omni software (Strategic Diagnostics, Inc.).

## 3. Results

### 3.1. Biodegradation Kinetics of Alkylated PAHs

**Figure 1:**
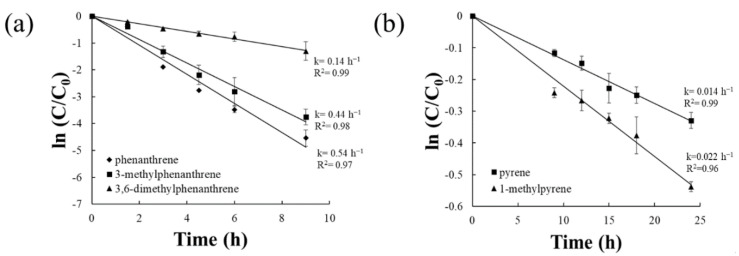
First-order kinetic fits of biodegradation experiments for (**a**) phenanthrene, 3-methylphenanthrene, and 3,6-dimethylphenanthrene, and (**b**) pyrene and 1-methylpyrene with the obtained rate constants (k). Error bars denote standard errors of triplicate analysis.

In each experiment, the final biomass was counted to ensure that the bacterial activity was maintained. Triplicate analysis showed that the initial and final biomasses of phenanthrenes were 2.54 (±0.72) × 10^7^ and 2.47 (±0.42) × 10^7^ cfu mL^−1^ and those of pyrenes were 5.77 (±0.41) × 10^7^ and 5.42 (±0.49) × 10^7^ cfu mL^−1^, demonstrating that the bacterial biomass was maintained during the test. No colonies were observed for sterilized controls. The sterilized controls were used to evaluate potential losses other than biodegradation including the sorption of chemicals to the glass surfaces and inactive biomass. The observed biodegradation of 5 chemicals followed first-order degradation kinetics (Figure 1). The biodegradation rate constants of phenanthrene and its alkyl derivatives were much higher than those of pyrene and 1-methylpyrene. As shown in Figure 1a, the biodegradation of phenanthrene (k = 0.54 ± 0.04 h^−1^) was fastest, followed by 3MPhe (k = 0.44 ± 0.05 h^−1^) and 36DMPhe (k = 0.14 ± 0.02 h^−1^). However, the biodegradation rate constant of 1MP (k = 0.022 ± 0.002 h^−1^) was higher than that of pyrene (k = 0.014 ± 0.001 h^−1^) (Figure 1b).

### 3.2. Fractionation of Transformation Products

During the experiments to determine the biodegradation rate constants of 36DMPhe and 1MP, new peaks with retention times shorter than those of the parent compounds were detected, and the areas of those unidentified peaks were increased in proportion to the degree of degradation of the original compounds in ultra-high pressure liquid chromatograph-fluorescence detector (UPLC-FLD) analysis (Figure S1, Supplementary Material).

To identify the transformation products that appeared in the UPLC-FLD chromatogram, their effluent fractions were enriched as explained in Section 2.4. As shown in the chromatograms of 36DMPhe (Figure S2a, Supplementary Material) and the sample after biodegradation (Figure S2b, Supplementary Material), the peak of 36DMPhe nearly completely disappeared, and two major new peaks of potential transformation products were identified after biodegradation. The column effluents containing the two peaks were collected separately and chromatograms for the isolated fractions (36DMPhe_F1 and 36DMPhe_F2) were obtained (Figure S2c,d, Supplementary Material). The transformation products were thought to be more polar than 36DMPhe because they showed shorter retention times during separation using a reverse-phase column [49]. The fluorescence emission of each unidentified peak was tested after excitation at 260 nm, and the optimal emission wavelength (λ_em_) was observed at 353.3 nm, which was the same optimum λ_em_ as that of 36DMPhe (Figure S3, Supplementary Material), suggesting that the conjugated phenyl ring structure was retained.

Figure S4 shows the chromatograms for (a) 1MP, (b) biodegradation mixture of 1MP after 24 h, and (c) the isolated fraction containing the peak of the transformation product in (b). As with the case of biotransformation products of 36DMPhe, transformation products of 1MP should be more polar than the parent compound. The optimal fluorescence emission wavelength (λ_em_) of unidentified peak was observed at 374.5 nm after excitation at 260 nm, which was same with the optimal emission wavelength of 1MP (Figure S5, Supplementary Material).

### 3.3. Identification of Transformation Products

Transformation products in the isolated fractions were identified by overlaying the GC-MS chromatograms obtained from the derivatized fractionated samples and fractionated controls without test chemicals. Because the metabolites in the isolated fractions are thought to be more polar than the parent compounds and to contain phenolic functional groups derivatized by a solution of *N*-*tert*-butyldimethylsilyl-*N*-methyltrifluoroacetamide with 1% *tert*-butyldimethylchlorosilane (MTBSTFA), it is expected that their retention times under the GC-MS separation conditions are longer than those of the parent compounds. Thus, observed peaks only in the isolated fractions were chosen as metabolite candidates. In Table 2, the mass spectrums and retention times of the tentative metabolites in the selected chromatograms for each fraction are shown with those of the parent compounds. Mass spectrum of three tentatively identified metabolites are also shown in Figure S6 (Supplementary Material).

For 36DMPhe_F2, the chromatogram at its retention time of 28.32 min was selected as the one of the metabolites. This peak was predicted to be a derivatized (6-methylphenanthrene-3-yl)methanol based on the molecular ion (M^+^) at *m/z* 336 and the major fragment ions at *m/z* 321 [M-CH_3_]^+^, 279 [M-C_4_H_9_]^+^, and 205 [M-C_6_H_15_SiO]^+^. Although no authentic standards were available for identification, the peak was confirmed by a mass shift of +14 (methyl substituted) of the major fragment ions relative to the fragment ions for 1-phenanthrenemethanol [22].

For 36DMPhe_F1, the peak at 37.35 min was predicted to be 6-methylphenanthrene-3-carboxylic acid according to the characteristics of the derivatized major fragment ions at *m/z* 335 [M-CH_3_]^+^, 293 [M-C_4_H_9_]^+^, 218.9 [M-C_6_H_15_SiO]^+^, and 191 [M-C_7_H_15_SiO_2_]^+^. Similar to the case of 36DMPhe_F2, the peak was confirmed based on the major fragment ions of 1-phenanthrene-carboxylic acid [22] and 4-phenanthrene-carboxylic acid [50,51,52] which showed a mass shift of +14 (methyl substitution).

The peak with its retention time of 32.522 min in the chromatograms of 1MP_F was identified as 1-pyrenemethanol by comparison with an authentic standard. The major fragment ions were *m/z* 346.1 [M]^+^, 289.1 [M-C_4_H_9_]^+^, and 215.0 [M-C_6_H_15_SiO]^+^. These mass fragments were identical to those of the standard with its retention time of 32.528 min (Figure S7, Supplementary Material).

During bacterial degradation, the transformation products become more polar and were more water-soluble than their parents [53,54]. Using EPISuite ver. 4.11 [45] adopting a group contribution method, the solubilities of the identified transformation products were estimated as 0.77, 15, and 12 μM for 36DMPhe_F1, 36DMPhe_F2, and 1MP_F, respectively, and the values were much higher than those of 3,6-DMPhe (0.18 μM) and 1-MP (0.46 μM).

### 3.4. Evaluation of Luminescence Inhibition by Transformation Products

**Figure 2:**
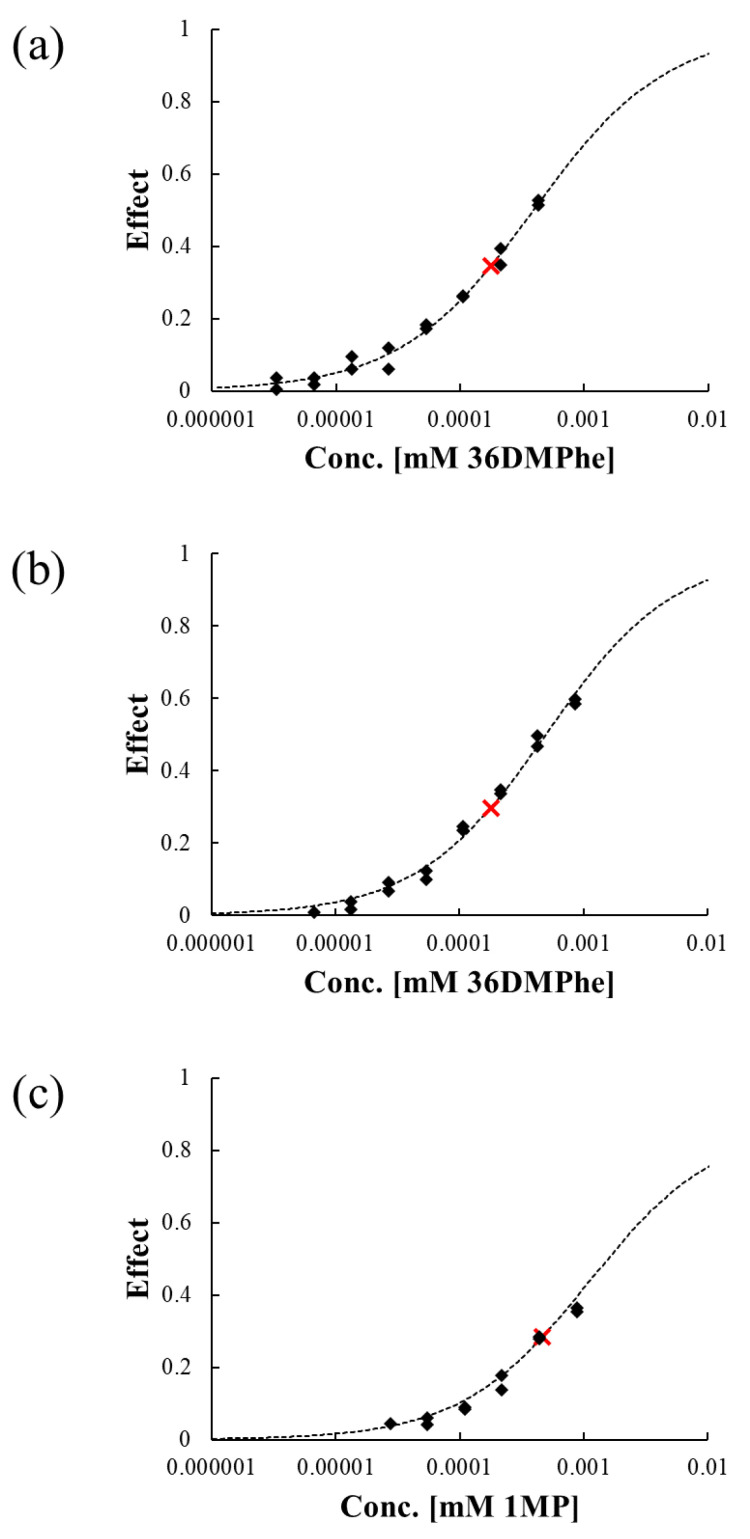
Dose-response curves for luminescence inhibition of *A. fischeri* by (**a**) 36DMP_F1, (**b**) 36DMP_F2, and (**c**) 1MP_F. The concentrations are expressed as the corresponding initial concentrations of their parent compounds. The black diamonds represent the experimental data and red cross marks represent luminescence inhibition of the isolated fractions at the water solubility of the parent compounds.

The toxicity of isolated fractions in Section 2.2 was evaluated by using the luminescence inhibition of *A. fischeri* as the toxicity endpoint (Figure 2). Although luminescence inhibition by 36DMPhe and 1MP was not observed at their water solubility limits in the previous study [47], clear dose-response relationships were observed for all three isolated fractions (Figure 2). Because the λ_em_ for the transformation products did not differ from those of the parent compounds, the levels of transformation products were estimated based on the relationship between the peak area and parent compound mass assuming that they had similar chromatophores.

As shown in Figure 2, the isolated fractions were more soluble in water than the parent compounds, and the observed luminescence inhibition at the solubility level of the parent compounds was greater (shown in cross marks) than those of parent, which were not observable [47]. At the solubility level of 36DMPhe, 34.6% and 29.7% luminescence inhibition was observed by 36DMPhe_F1 and 36DMPhe_F2, respectively. For 1MP_F, 28.3% luminescence inhibition was observed at the solubility level of 1MP.

## 4. Discussion

The biodegradation rate constants of (alkylated) phenanthrenes were much faster than (alkylated) pyrenes. This tendency was also observed in a previous study comparing the biodegradation of phenanthrene, 1-methylphenanthrene, 2-methylphenanthrene, 3,6DMPhe, pyrene, and 1MP by *S. quisquiliarum* (DSM 7526), showing a higher biodegradation rate in phenanthrene and their alkylated forms than that for pyrenes [24].

Earlier studies showed conflicting effects of alkylation of phenanthrene and pyrene on the biodegradation rate constants. Whereas the biodegradation rate of 1-methylphenanthrene by *Sphingobium* sp. MP9-4 was slower than that of phenanthrene [22], the rate constants were increased by methylation for 1-methylphenanthrene (3.24 ± 1.44 L mg_protein_^−1^ h^−1^) and 2-methylphenanthrene (3.73 ± 1.56 L mg_protein_^−1^ h^−1^) and decreased for 36DMPhe (1.56 ± 0.36 L mg_protein_^−1^ h^−1^) compared to unsubstituted phenanthrene (2.09 ± 0.79 L mg_protein_^−1^ h^−1^) [24].

For pyrene and 1-methylpyrene, the literature values are 1.10 ± 0.31 L mg_protein_^−1^ h^−1^ and 0.82 ± 0.50 L mg_protein_^−1^ h^−1^, respectively [24], indicating no significant difference between the biodegradation rate constants of these compounds. The biodegradation rate constant decreased with alkylation for phenanthrenes whereas the rate constant for 1-methylpyrene was greater than that of pyrene in this study. It has been suggested that the main site for dioxygenation of phenanthrene by *Sphingomonas* sp. is the 5,6-C site (or 3,4-C site which are equivalent; Figure S8a, Supplementary Material) [22]. In 3-methylphenanthrene, however, the 3,4-C site is hindered by methylation, resulting in inhibition of dioxygenation-derived metabolism. In 3,6-dimethylphenanthrene, both dioxygenation sites are hindered by methyl groups, resulting in a significant decrease in the biodegradation rate constants compared to the unsubstituted form. The observed trend in the biodegradation kinetics for phenanthrene, 3-methylphenanthrene, and 3,6-dimethylphenanthrene agrees well with this hypothesis. In case of pyrene, no site preference was observed in the metabolism because of its high symmetry (Figure S8b, Supplementary Material). Diverse metabolic pathways by methylation of pyrene may result in a higher biodegradation rate constant of 1MP than that of pyrene. The identification of the biotransformation products of 36DMPhe and 1MP also supported this hypothesis as explained below. However, it is also well-known that co-factors such as metal ions included in the culture medium influence on the observed bacterial or enzymatic degradation rate and transformation pathways [55,56]. Further investigations on the mechanism and pathways of the transformation of alkylated PAHs would explain the differences in biodegradation rates among PAHs and their alkylated homologues.

Two isolated fractions for the metabolic products of 36DMPhe were identified as monooxygenated form of 36DMPhe (36DMPhe_F2) and monooxygenated form of 36DMP_F2 (36DMPhe_F1), respectively. This indicates that monooxygenation of the methyl group could initiate the degradation of 36DMPhe by *S*. *quisquiliarum*. In bacterial degradation of 1MP by the strain MP9–4, monooxygenation of the methyl group occurred with dioxygenation on carbon atoms in benzene ring simultaneously [22]. It was observed that unsubstituted positions in benzene ring was preferentially attacked in bacterial degradation of 1-methylphenanthrene [22]. For 36DMPhe in this study, however, monooxygenation of the methyl group appeared to be the major biodegradation pathway because the 5,6-C site (or equivalent 3,4-C site), which is known as the main site for dioxygenation by *Sphingobium* sp. [22], is hindered by methylation. The isolated fraction of the metabolic products of 1MP, 1MP_F, was also identified as the monooxygenated form of 1MP, confirming that monooxygenation of the methyl group also occurred during bacterial degradation of 1MP. This indicates that the alkyl-substitution on pyrene could serve multiple degradation pathways and make the biodegradation faster than unsubstituted pyrene.

The luminescence inhibitions of *A. fischeri* by the isolated fractions from biodegradation products of 36DMPhe and 1MP were detectable despite non-observable toxicity for their original compounds, indicating that the ecotoxicity of alkylated PAHs could be enhanced via biodegradation processes. This increase in toxicity can be explained by the increased solubility of the compounds via biotransformation or by intrinsic toxicity of transformation products. The median effect concentrations (EC_50_) of the identified transformation products were derived using quantitative structure-activity relationships developed for predicting the baseline toxicity of petroleum hydrocarbons in our previous study [57] with log K_ow_ values estimated by EPISuite^TM^. These values were 0.39, 0.49 and 1.5 μM for 36DMPhe_F1, 36DMPhe_F2, and 1MP_F, respectively. The toxicity ratios (TR) were calculated by dividing the EC_50_ values derived from the quantitative structure-activity relationships prediction by the measured EC_50_ in this study. These values were 0.47, 4.61, and 1.23 for 36DMPhe_F1, 36DMPhe_F2, and 1MP_F, respectively. It is generally accepted that chemicals with a TR < 5 cause narcosis [58]. Although all tested fractions could be classified as baseline toxicants, the fraction 36DMPhe_F2 may have a specific toxic mode-of-action. The luminescence inhibition of *A. fischeri* by aromatic acids showed that the toxicity increased with increasing hydrophobicity, which is the general tendency observed for baseline toxicants [33]. Previous studies compared the toxicity of PAHs and their oxygenated derivatives [35,36]. Enhanced chronic toxicity in the early life stage of *Oryzias latipes* was observed for 1-methylphenanthrene by hydroxylation, as hydroxylated 1-methylphenanthrene derivatives were 4-fold more toxic than 1-methylphenanthrene [35]. The developmental toxicity of chrysene and its hydroxylated derivatives tested using zebrafish embryos showed similar results, indicating that the photochemical or microbial transformation of PAHs enhances the toxicity of PAHs [36]. This suggests that both the toxic potency and efficacy of metabolic products of alkylated PAHs could be greater than their parent compounds because of the greater inherent toxicity of metabolites and/or enhanced water solubility.

## 5. Conclusions

The present study focused on the microbial degradation of alkylated PAHs by *S. quisquiliarum* and evaluation of the ecotoxicity for their metabolites with the identification. The biodegradation kinetics of (alkylated) phenanthrenes and pyrenes were determined, and the biodegradation rates of phenanthrenes were much faster than pyrenes. The identification of the isolated fractions for the metabolic products of 36DMPhe and 1MP showed that the monooxygenation of methyl groups was one of the main metabolic pathways of biodegradation of alkylated PAHs. The alkyl-substitution made the biodegradation PAHs faster or slower comparing to the unsubstituted PAHs by serving multiple degradation pathways or hindrance of dioxygenation on the non-methylated benzene ring. The luminescence inhibitions of *A. fischeri* by 36DMPhe and 1MP were detectable despite non-observable toxicity for their original compounds, indicating the ecotoxicity of alkylated PAHs could be enhanced by biodegradation process. This study suggests that both toxic potency and efficacy are increased during the biodegradation of alkylated PAHs, particularly under a constant supply of the parent alkylated PAHs after spills. Although a few strains which can degrade alkylated PAHs may be less vulnerable, the meta-stable oxygenated products may be more toxic and act as important transient contaminants of oil spill sites. Further studies are needed to determine the time-course changes in toxicity of alkylated PAHs during weathering processes after oil spills to improve risk characterization.

## Figures and Tables

**Table 1:** Chemical structure, molecular weight (MW), aqueous solubility (S), the initial concentration in biodegradation tests (C_initial_), logarithm of octanol-water partition coefficient (log K_ow_), and lipid-water partition coefficient (log K_lipw_) of selected (alkylated) polycyclic aromatic hydrocarbons (PAHs).

Chemical	Structure	MW (g mol^−1^)	S (mg L^−1^ at 25 °C)	C_ini_ (mg L^−1^, mean ± SD)	log K_ow_^1^	log K_lipw_
phenanthrene	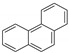	178	0.82 ^2^	0.72 ± 0.03	4.46	5.05 ^4^
3MPhe	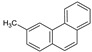	192.26	0.63 ^3^	0.56 ± 0.02	4.89	5.24 ^3^
36DMPhe	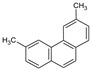	206.29	0.037 ^3^	0.027 ± 0.001	5.44	5.58 ^3^
Pyrene	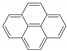	202	0.086 ^2^	0.088 ± 0.003	5.18	5.74 ^4^
1MP	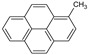	216.28	0.10 ^3^	0.102 ± 0.005	5.48	5.76 ^3^

^1^ Predicted data using the KOWWIN v1.68 in EPI Suite v4.11 (Ref [45]), ^2^ Ref [46], ^3^ Ref [47], ^4^ Ref [48].

**Table 2:** Retention times and mass fragmentation spectrums of 3,6-dimethylphenanthrene, 1-methylpyrene, and tentatively identified metabolic products in the fractions, 3,6DMPhe_F1, 3,6DMPhe_F2, and 1MP_F.

Chemical	Retention Time (min)	Mass Spectrum (*m*/*z*)
3,6DMPhe	16.79	206.1, 202.1, 191.1, 187.0
36DMPhe_F1	37.35	335.0, 293.0, 218.9, 204.0, 190.9, 183.8
36DMPhe_F2	28.32	336.1, 321.0, 279.1, 220.1, 205.0, 189.0
1MP	22.02	216.1, 207.1, 199.9, 189.0, 163.0
1MP_F	32.52	346.1, 289.1, 215.0, 200.0, 188.9, 133.9

## Supplementary Materials

The following are available online at https://www.mdpi.com/1660-4601/17/17/6416/s1, Figure S1: Relative peak areas of (a) 3,6-dimethylphenanthrene and generated peak and (b) 1-methylpyrene and generated peak during biodegradation tests, Figure S2: HPLC chromatograms of (a) 3,6-dimethylphenanthrene, (b) products of biodegradation, (c) the first fractionated metabolic product (36DMPhe_F1) and (d) the second fractionated metabolic product (36DMPhe_F2), Figure S3: Three-dimensional scanning of λ_em_ with fixed λ_ex_ of 260 nm (left) and the optimum λ_em_ (right) for (a) 3,6-dimethylphenanthrene and (b) the first fractionated metabolic product (36DMPhe_F1) and (c) the second fractionated metabolic product (36DMPhe_F2), Figure S4: HPLC chromatograms of (a) 1-methylpyrene, (b) products of biodegradation, (c) the fractionated metabolic product (1MP_F), Figure S5: Three-dimensional scanning of λem with fixed λex of 260 nm (left panel) and the optimum λem (right panel) for (a) 1-methylpyrene, (b) the fractionated metabolic product (1MP_F), Figure S6: The mass fragmentation spectrum of the identified metabolic products of (a) 3,6DMPhe_F1, (b) 3,6DMPhe_F2, and (c) 1MP_F with their derivatized structures, Figure S7: The mass fragmentation spectrum of an authentic standard of 1-pyrenemethanol with the derivatized structure, Figure S8: The chemical structures of (a) phenanthrene and (b) pyrene with their carbon numbering.

## References

[1] Baklanov A., Hänninen O., Slørdal L.H., Kukkonen J., Bjergene N., Fay B., Finardi S., Hoe S.C., Jantunen M., Karppinen A. (2007). Integrated systems for forecasting urban meteorology, air pollution and population exposure. Atmos. Chem. Phys..

[2] Latimer J.S., Zheng J. (2003). The Sources, Transport, and Fate of PAHs in the Marine Environment. PAHs: An Ecotoxicological Perspective.

[3] Abdel-Shafy H.I., Mansour M.S.M. (2016). A review on polycyclic aromatic hydrocarbons: Source, environmental impact, effect on human health and remediation. Egypt J. Pet..

[4] Hawthorne S.B., Miller D.J., Kreitinger J.P. (2006). Measurement of total polycyclic aromatic hydrocarbon concentrations in sediments and toxic units used for estimating risk to benthic invertebrates at manufactured gas plant sites. Environ. Toxicol. Chem..

[5] Kang H.-J., Lee S.-Y., Roh J.-Y., Yim U.H., Shim W.J., Kwon J.-H. (2014). Prediction of ecotoxicity of heavy crude oil: Contribution of measured components. Environ. Sci. Technol..

[6] Ramseur J.L. (2010). CRS Report for Congress Deepwater Horizon Oil Spill: The Fate of the Oil.

[7] Camilli R., Reddy C.M., Yoerger D.R., Van Mooy B.A.S., Jakuba M.V., Kinsey J.C., McIntyre C.P., Sylva S.P., Maloney J.V. (2010). Tracking hydrocarbon plume transport and biodegradation at deepwater horizon. Science.

[8] Hazen T.C., Dubinsky E.A., DeSantis T.Z., Andersen G.L., Piceno Y.M., Singh N., Jansson J.K., Probst A., Borglin S.E., Fortney J.L. (2010). Deep-sea oil plume enriches indigenous oil-degrading bacteria. Science.

[9] Seo J.-S., Keum Y.-S., Li Q.X. (2010). Bacterial degradation of aromatic compounds. Int. J. Environ. Res. Public Health.

[10] Cerniglia C.E. (1992). Biodegradation of polycyclic aromatic hydrocarbons. Biodegradation.

[11] Waigi M.G., Kang F., Goikavi C., Ling W., Gao Y. (2015). Phenanthrene biodegradation by sphingomonads and its application in the contaminated soils and sediments: A review. Int. Biodeterior. Biodegrad..

[12] Peng R.H., Xiong A.S., Xue Y., Fu X.Y., Gao F., Zhao W., Tian Y.S., Yao Q.H. (2008). Microbial biodegradation of polyaromatic hydrocarbons. FEMS Microbiol. Rev..

[13] Haritash A.K., Kaushik C.P. (2009). Biodegradation aspects of polycyclic aromatic hydrocarbons (PAHs): A review. J. Hazard Mater.

[14] Rojo-nieto E., Perales-Vargas-Machuca J.A., Singh S.N. (2012). Microbial Degradation of PAHs: Organisms and Environmental Compartments. Microbial Degradation of Xenobiotics.

[15] Ghosal D., Ghosh S., Dutta T.K., Ahn Y. (2016). Current state of knowledge in microbial degradation of polycyclic aromatic hydrocarbons (PAHs): A review. Front. Microbiol..

[16] Basu A., Dixit S.S., Phale P.S. (2003). Metabolism of benzyl alcohol via catechol ortho-pathway in methylnaphthalene-degrading *Pseudomonas putida* CSV86. Appl. Microbiol. Biotechnol..

[17] Mahajan M.C., Phale P.S., Vaidyanathan C.S. (1994). Evidence for the involvement of multiple pathways in the biodegradation of 1- and 2-methylnaphthalene by *Pseudomonas putida* CSV86. Arch. Microbiol..

[18] Musat F., Galushko A., Jacob J., Widdel F., Kube M., Reinhardt R., Wilkes H., Schink B., Rabus R. (2009). Anaerobic degradation of naphthalene and 2-methylnaphthalene by strains of marine sulfate-reducing bacteria. Environ. Microbiol..

[19] Saftić S., Fedorak P.M., Andersson J.T. (1993). Transformations of methyldibenzothiophenes by three *Pseudomonas* isolates. Environ. Sci. Technol..

[20] Dutta T.K., Selifonov S.A., Gunsalus I.C. (1998). Oxidation of methyl-substituted naphthalenes: Pathways in a versatile *Sphingomonas paucimobilis* strain. Appl. Environ. Microbiol..

[21] Tøndervik A., Bruheim P., Berg L., Ellingsen T.E., Kotlar H.K., Valla S., Throne-Holst M. (2012). *Ralstonia* sp. U2 naphthalene dioxygenase and *Comamonas* sp. JS765 nitrobenzene dioxygenase show differences in activity towards methylated naphthalenes. J. Biosci. Bioeng..

[22] Zhong J., Luo L., Chen B., Sha S., Qing Q., Tam N.F.Y., Zhang Y., Luan T. (2017). Degradation pathways of 1-methylphenanthrene in bacterial *Sphingobium* sp. MP9-4 isolated from petroleum-contaminated soil. Mar. Pollut. Bull.

[23] Wammer K.H., Peters C.A. (2005). Polycyclic aromatic hydrocarbon biodegradation rates: A structure-based study. Environ. Sci. Technol..

[24] Dimitriou-Christidis P., Autenrieth R.L., McDonald T.J., Desai A.M. (2007). Measurement of biodegradability parameters for single unsubstituted and methylated polycyclic aromatic hydrocarbons in liquid bacterial suspensions. Biotechnol. Bioeng..

[25] Desai A.M., Autenrieth R.L., Dimitriou-Christidis P., McDonald T.J. (2008). Biodegradation kinetics of select polycyclic aromatic hydrocarbon (PAH) mixtures by *Sphingomonas paucimobilis* EPA505. Biodegradation.

[26] Knightes C.D., Peters C.A. (2003). Aqueous phase biodegradation kinetics of 10 PAH compounds. Environ. Eng. Sci..

[27] Siddiqi M.A., Yuan Z.X., Honey S.A., Kumar S., Sikka H.C. (2002). Metabolism of PAHs and methyl-substituted PAHs by *Sphingomonas paucimobilis* strain EPA 505. Polycycl. Aromat. Compd..

[28] Pampanin D.M., Sydnes M.O., Kutcherov V., Kolesnikov A. (2013). Chapter 5: Polycyclic Aromatic Hydrocarbons a Constituent of Petroleum: Presence and Influence in the Aquatic Environment. Hydrocarbon.

[29] Yim U.H., Ha S.Y., An J.G., Won J.H., Han G.M., Hong S.H., Kim M., Jung J.H., Shim W.J. (2011). Fingerprint and weathering characteristics of stranded oils after the Hebei Spirit oil spill. J. Hazard Mater..

[30] Neff J., Lee K., DeBlois E.M., Lee K., Neff J. (2011). Produced Water: Overview of Composition, Fates, and Effects. Produced Water: Environmental Risks and Advances in Mitigation Technologies.

[31] Lundstedt S., White P.A., Lemieux C.L., Lynes K.D., Lambert I.B., Oberg L., Haglund P., Tysklind M. (2007). Sources, fate, and toxic hazards of oxygenated polycyclic aromatic hydrocarbons (PAHs) at PAH-contaminated sites. Ambio.

[32] Arp H.P.H., Lundstedt S., Josefsson S., Cornelissen G., Enell A., Allard A.S., Kleja D.B. (2014). Native oxy-PAHs, N-PACs, and PAHs in historically contaminated soils from Sweden, Belgium, and France: Their soil-porewater partitioning behavior, bioaccumulation in *Enchytraeus crypticus*, and bioavailability. Environ. Sci. Technol..

[33] Frank R.A., Kavanagh R., Burnison B.K., Arsenault G., Headley J.V., Peru K.M., Van Der Kraak G., Solomon K.R. (2008). Toxicity assessment of collected fractions from an extracted naphthenic acid mixture. Chemosphere.

[34] Jones D., Scarlett A.G., West C.E., Rowland S.J. (2011). Toxicity of individual naphthenic acids to *Vibrio fischeri*. Environ. Sci. Technol..

[35] Fallahtafti S., Rantanen T., Brown R.S., Snieckus V., Hodson P.V. (2012). Toxicity of hydroxylated alkyl-phenanthrenes to the early life stages of Japanese medaka (*Oryzias latipes*). Aquat. Toxicol..

[36] Diamante G., do Amaral e Silva Müller G., Menjivar-Cervantes N., Xu E.G., Volz D.C., Dias Bainy A.C., Schlenk D. (2017). Developmental toxicity of hydroxylated chrysene metabolites in zebrafish embryos. Aquat. Toxicol..

[37] Kawano M., Uno S., Koyama J., Kokushi E., McElroy A. (2017). Effects of oxygenated polycyclic aromatic hydrocarbons on the early life stages of Japanese medaka. Environ. Sci. Pollut. Res..

[38] Dogra Y., Scarlett A.G., Rowe D., Galloway T.S., Rowland S.J. (2018). Predicted and measured acute toxicity and developmental abnormalities in zebrafish embryos produced by exposure to individual aromatic acids. Chemosphere.

[39] Wincent E., Jönsson M.E., Bottai M., Lundstedt S., Dreij K. (2015). Aryl hydrocarbon receptor activation and developmental toxicity in zebrafish in response to soil extracts containing unsubstituted and oxygenated pahs. Environ. Sci. Technol..

[40] Roh J.-Y., Lee H., Kwon J.-H. (2014). Changes in the expression of *cyp35a* family genes in the soil nematode *Caenorhabditis elegans* under controlled exposure to chlorpyrifos using passive dosing. Environ. Sci. Technol..

[41] Kwon J.-H., Wuethrich T., Mayer P., Escher B.I. (2009). Development of a dynamic delivery method for in vitro bioassays. Chemosphere.

[42] Smith K.E.C., Rein A., Trapp S., Mayer P., Karlson U.G. (2012). Dynamic passive dosing for studying the biotransformation of hydrophobic organic chemicals: Microbial degradation as an example. Environ. Sci. Technol..

[43] Stanier R.Y., Palleroni N.J., Doudoroff M. (1966). The aerobic Pseudomonads: A Taxonomic study. J. Gen. Microbiol..

[44] Cohen-Bazire G., Sistrom W.R., Stanier R.Y. (1957). Kinetic studies of pigment synthesis by non-sulfur purple bacteria. J. Cell Comp. Physiol..

[45] US Environmental Protection Agency (2012). EPIsuite^TM^ v.4.11. https://www.epa.gov/tsca-screening-tools/download-epi-suitetm-estimation-program-interface-v411.

[46] Kwon H.-C., Kwon J.-H. (2012). Measuring aqueous solubility in the presence of small cosolvent volume fractions by passive dosing. Environ. Sci. Technol..

[47] Kang H.-J., Lee S.-Y., Kwon J.-H. (2016). Physico-chemical properties and toxicity of alkylated polycyclic aromatic hydrocarbons. J. Hazard Mater..

[48] van der Heijden S.A., Jonker M.T.O. (2009). Evaluation of liposome-water partitioning for predicting bioaccumulation potential of hydrophobic organic chemicals. Environ. Sci. Technol..

[49] Malmquist L.M.V., Selk H., Jørgensen K.B., Christensen J.H. (2015). Polycyclic aromatic acids are primary metabolites of alkyl-PAHs—A case study with *Nereis Diversicolor*. Environ. Sci. Technol..

[50] Ghosh I., Jasmine J., Mukherji S. (2014). Biodegradation of pyrene by a *Pseudomonas aeruginosa* strain RS1 isolated from refinery sludge. Bioresour. Technol..

[51] Vila J., López Z., Sabaté J., Minguillón C., Solanas A.M., Grifoll M. (2001). Identification of a novel metabolite in the degradation of pyrene by *Mycobacterium* sp. strain AP1: Actions of the isolate on two- and three-ring polycyclic aromatic hydrocarbons. Appl. Environ. Microbiol..

[52] Zhong Y., Luan T., Zhou H., Lan C., Tam N.F.Y. (2006). Metabolite production in degradation of pyrene alone or in a mixture with another polycyclic aromatic hydrocarbon by *Mycobacterium* sp.. Environ. Toxicol. Chem..

[53] Pagnout C., Rast C., Veber A.-M., Poupin P., Férard J.-F. (2006). Ecotoxicological assessment of PAHs and their dead-end metabolites after degradation by *Mycobacterium* sp. strain SNP11. Ecotoxicol. Environ. Saf..

[54] Šepič E., Bricelj M., Leskovšek H. (2003). Toxicity of fluoranthene and its biodegradation metabolites to aquatic organisms. Chemosphere.

[55] Singh S.N., Kumari B., Upadhyay S.K., Mishra S., Kumar D. (2013). Bacterial degradation of pyrene in minimal salt medium mediated by catechol dioxygenases: Enzyme purification and molecular size determination. Bioresour. Technol..

[56] Kumar S., Upadhayay S.K., Kumari B., Tiwari S., Singh S.N., Singh P.K. (2011). In vitro degradation of fluoranthene by bacteria isolated from petroleum sludge. Bioresour. Technol..

[57] Lee S.-Y., Kang H.-J., Kwon J.-H. (2013). Toxicity cutoff of aromatic hydrocarbons for luminescence inhibition of *Vibrio fischeri*. Ecotoxicol. Environ. Saf..

[58] Verhaar H.J.M., van Leeuwen C.J., Hermens J.L.M. (1992). Classifying environmental pollutants. 1. Structure-activity relationships for prediction of aquatic toxicity. Chemosphere.

