# Supplementary material for: Enhancement of Toxic Efficacy of Alkylated Polycyclic Aromatic Hydrocarbons Transformed by Sphingobium quisquiliarum
Source: Int J Environ Res Public Health. 2020 Sep 3;17(17):6416. doi: 10.3390/ijerph17176416 (PMC7503419; doi:10.3390/ijerph17176416)
Supplement: Supplementary file 1 [file ijerph-17-06416-s001.pdf]

Supplementary Material

Enhancement of toxic efficacy of alkylated polycyclic aromatic hydrocarbons transformed by *Sphingobium quisquiliarum*

*So-Young Lee and Jung-Hwan Kwon**

Division of Environmental Science and Ecological Engineering, Korea University, 145 Anam-ro, Seongbuk-gu, Seoul 02841, Republic of Korea

*Corresponding author:

Phone: +82 2 3290 3041

Fax: +82 2 953 0737

E-mail: junghwankwon@korea.ac.kr

ORCID: 0000-0002-6341-7562

Contents:

Figure S1. Relative peak areas of (a) 3,6-dimethylphenanthrene and generated peak and (b) 1-methylpyrene and generated peak during biodegradation tests.

Figure S2. HPLC Chromatograms of (a) 3,6-dimethylphenanthrene, (b) products of biodegradation, (c) the first fractionated metabolic product (36DMPhe_F1) and (d) the second fractionated metabolic product (36DMPhe_F2).

Figure S3. Three-dimensional scanning of λ_{em} with fixed λ_{ex} of 260 nm (left) and the optimum λ_{em} (right) for (a) 3,6-dimethylphenanthrene and (b) the first fractionated metabolic product (36DMPhe_F1) and (c) the second fractionated metabolic product (36DMPhe_F2).

Figure S4. HPLC chromatograms of (a) 1-methylpyrene, (b) products of biodegradation, (c)

the fractionated metabolic product (1MP_F).

Figure S5. Three-dimensional scanning of λ_{em} with fixed λ_{ex} of 260 nm (left panel) and the optimum λ_{em} (right panel) for (a)1-methylpyrene, (b) the fractionated metabolic product (1MP_F).

Figure S6. The mass fragmentation spectrum of the identified metabolic products of (a) 3,6DMPhe_F1, (b) 3,6DMPhe_F2, and (c) 1MP_F with their derivatized structures.

Figure S7. The mass fragmentation spectrum of an authentic standard of 1-pyrenemethanol with the derivatized structure.

Figure S8. The chemical structures of (a) phenanthrene and (b) pyrene with their carbon numbering.

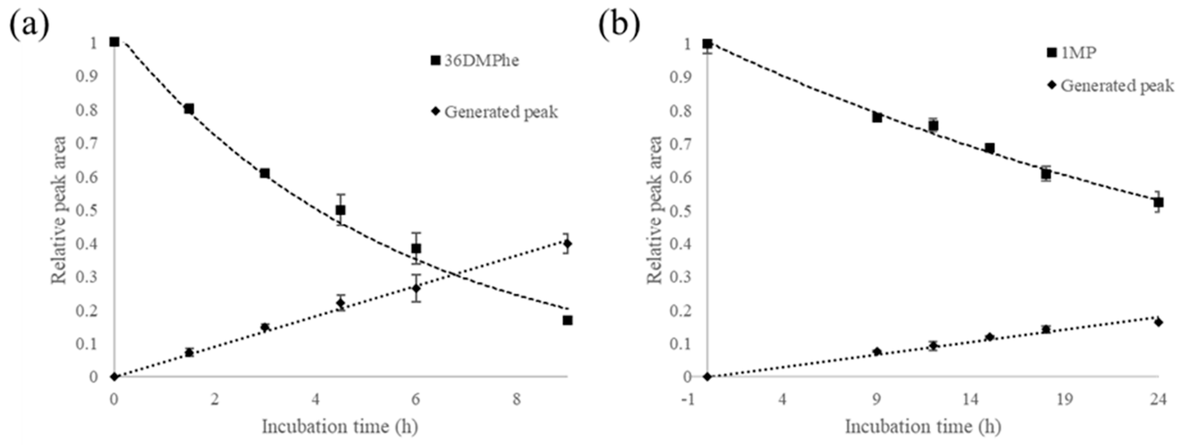

Figure S1. Relative peak areas of (a) 3,6-dimethylphenanthrene and generated peak and (b) 1-methylpyrene and generated peak during biodegradation tests.

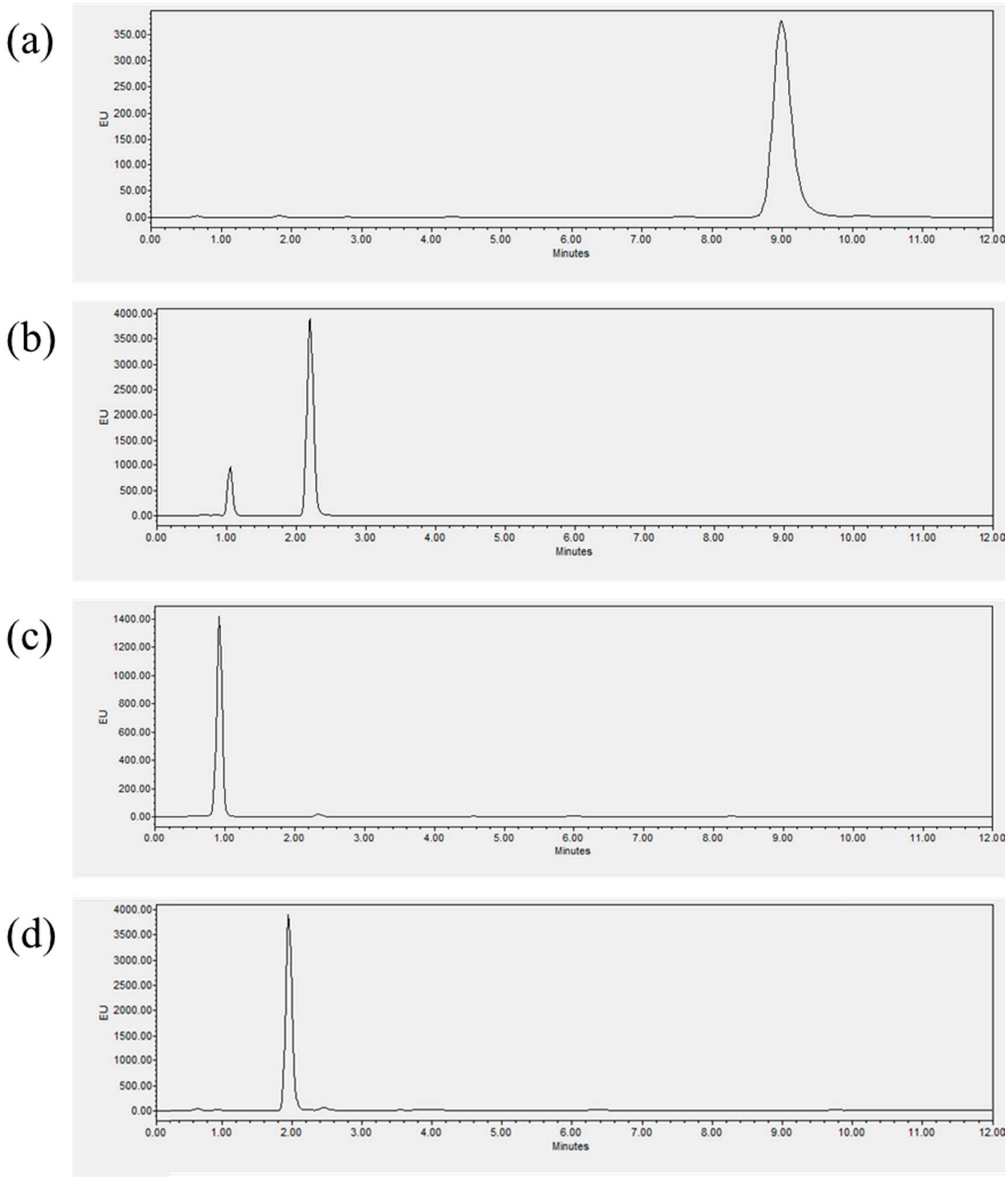

Figure S2. HPLC Chromatograms of (a) 3,6-dimethylphenanthrene, (b) products of biodegradation, (c) the first fractionated metabolic product (36DMPhe_F1) and (d) the second fractionated metabolic product (36DMPhe_F2).

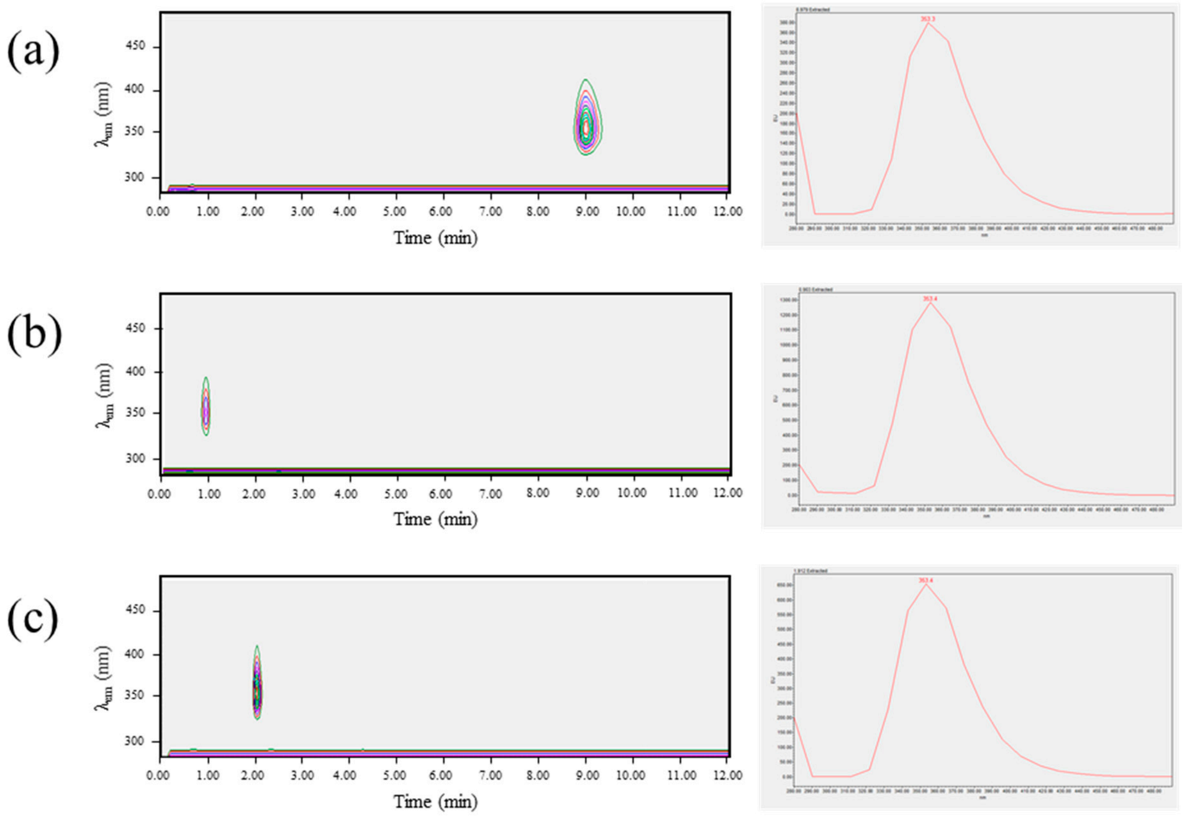

Figure S3. Three-dimensional scanning of λ_{em} with fixed λ_{ex} of 260 nm (left) and the optimum λ_{em} (right) for (a) 3,6-dimethylphenanthrene and (b) the first fractionated metabolic product (36DMPhe_F1) and (c) the second fractionated metabolic product (36DMPhe_F2).

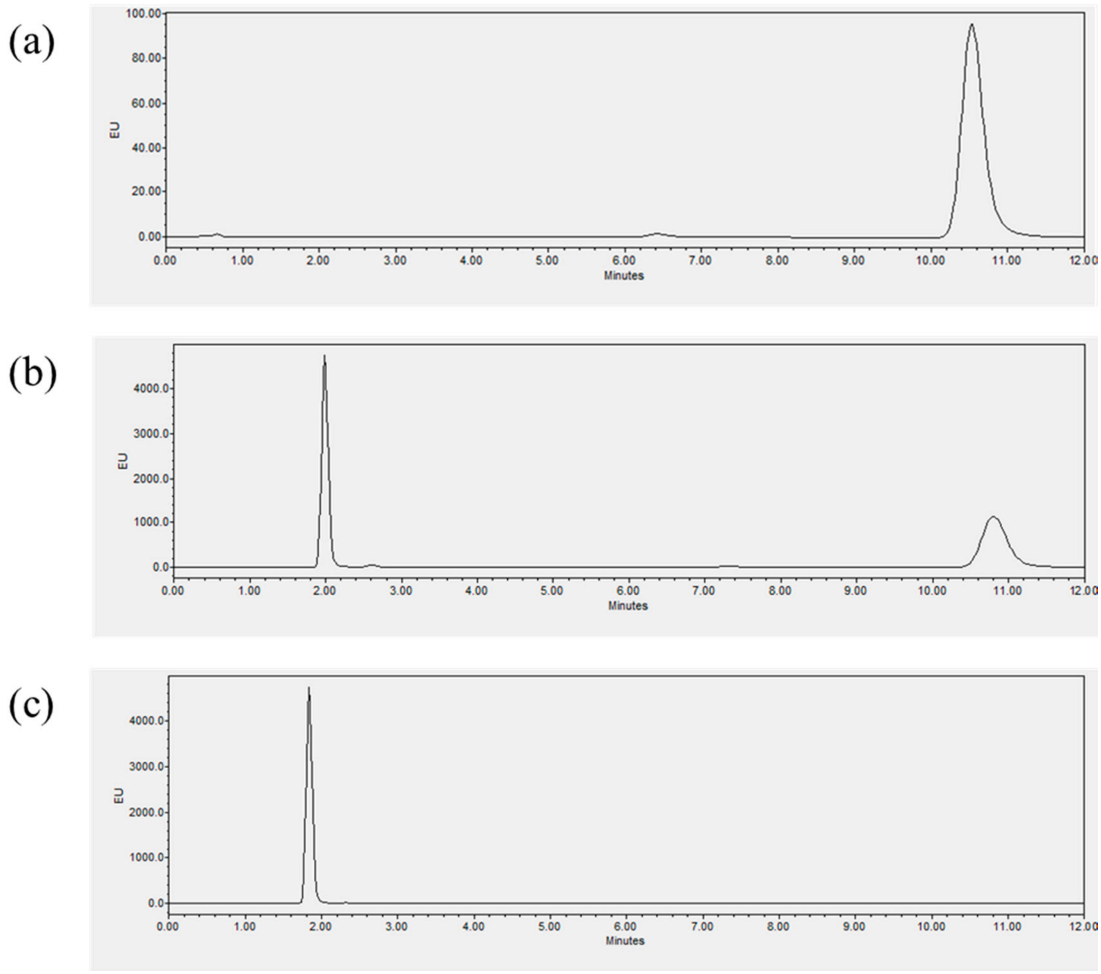

Figure S4. HPLC chromatograms of (a) 1-methylpyrene, (b) products of biodegradation, (c) the fractionated metabolic product (1MP_F).

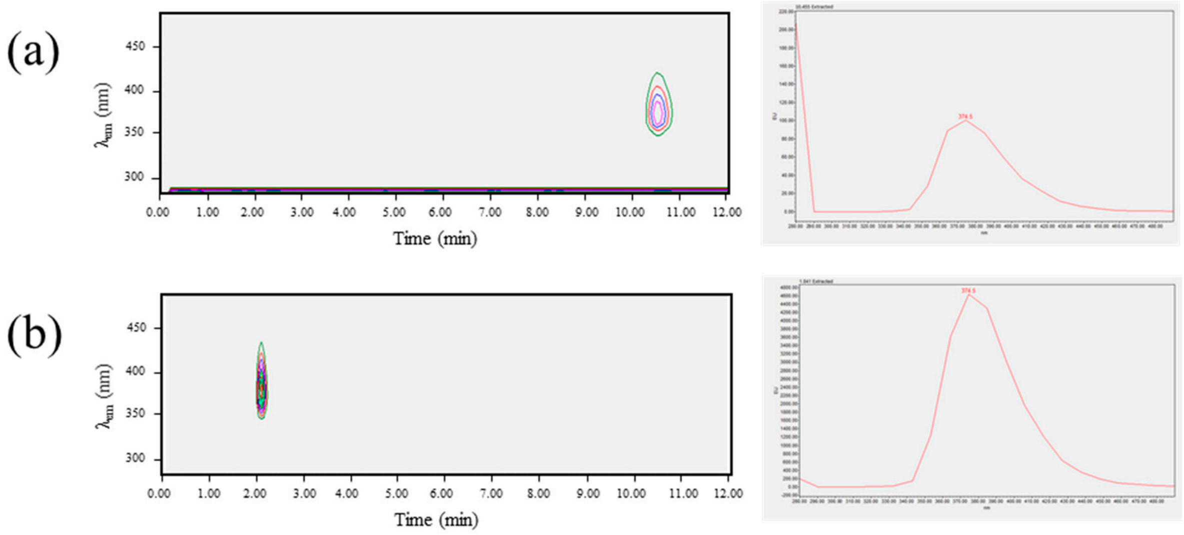

Figure S5. Three-dimensional scanning of λ_{em} with fixed λ_{ex} of 260 nm (left panel) and the optimum λ_{em} (right panel) for (a) 1-methylpyrene, (b) the fractionated metabolic product (1MP_F).

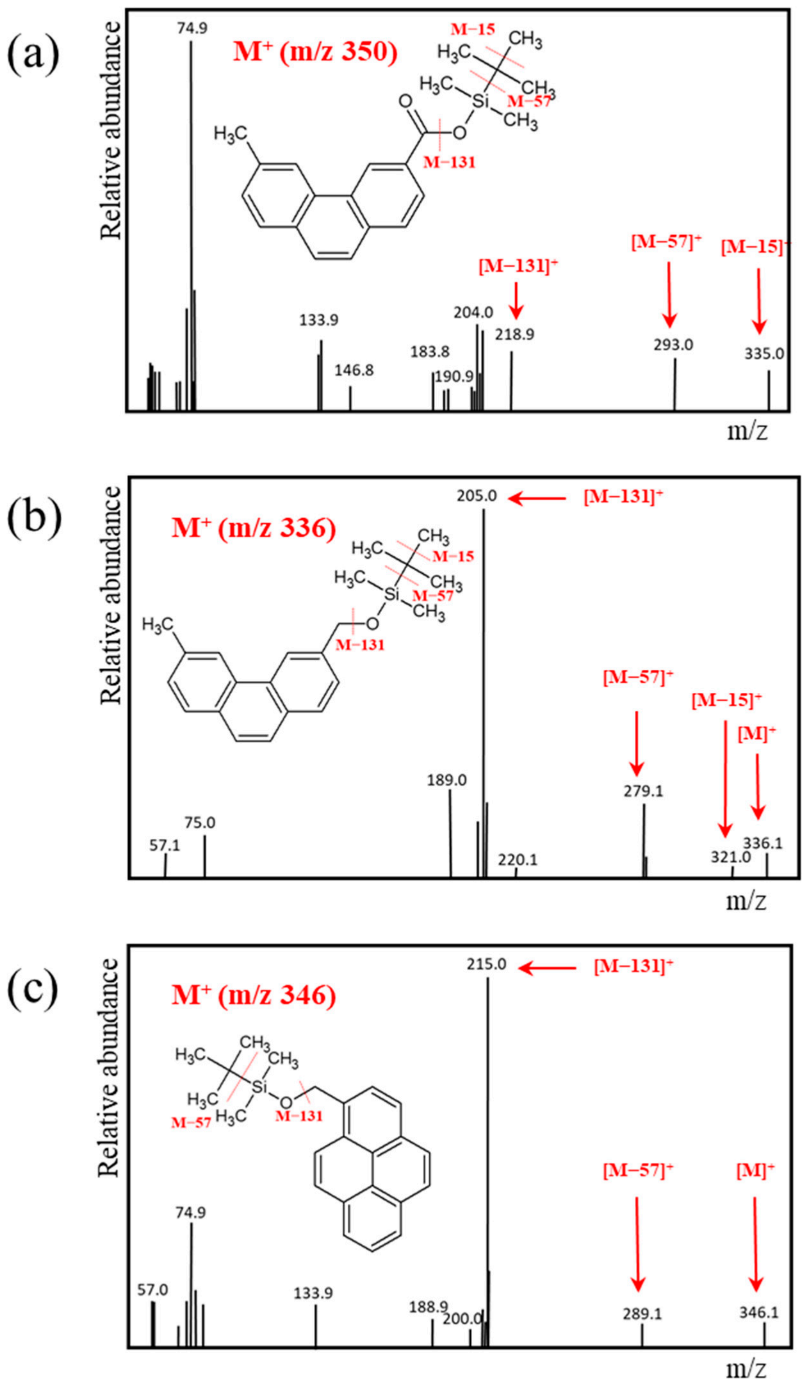

Figure S6. The mass fragmentation spectrum of the identified metabolic products of (a) 3,6DMPhe_F1, (b) 3,6DMPhe_F2, and (c) 1MP_F with their derivatized structures.

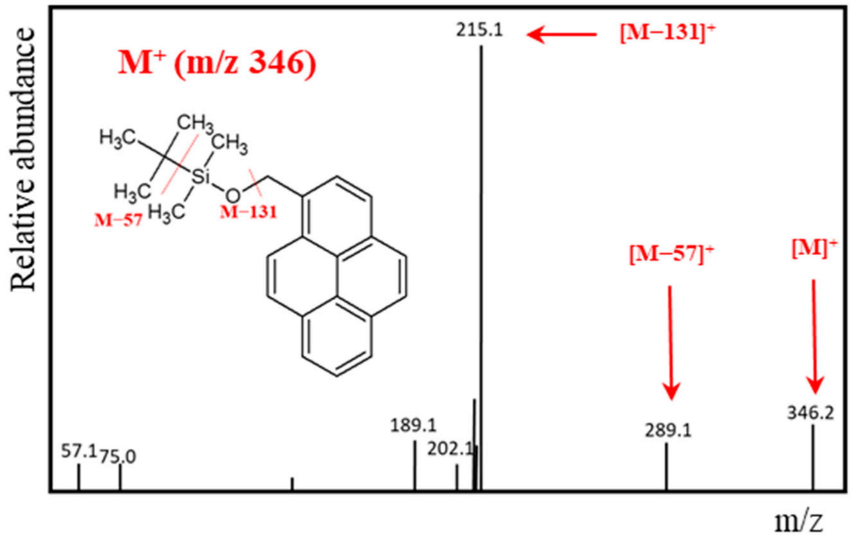

20

21 **Figure S7.** The mass fragmentation spectrum of an authentic standard of 1-pyrenemethanol

22 with the derivatized structure.

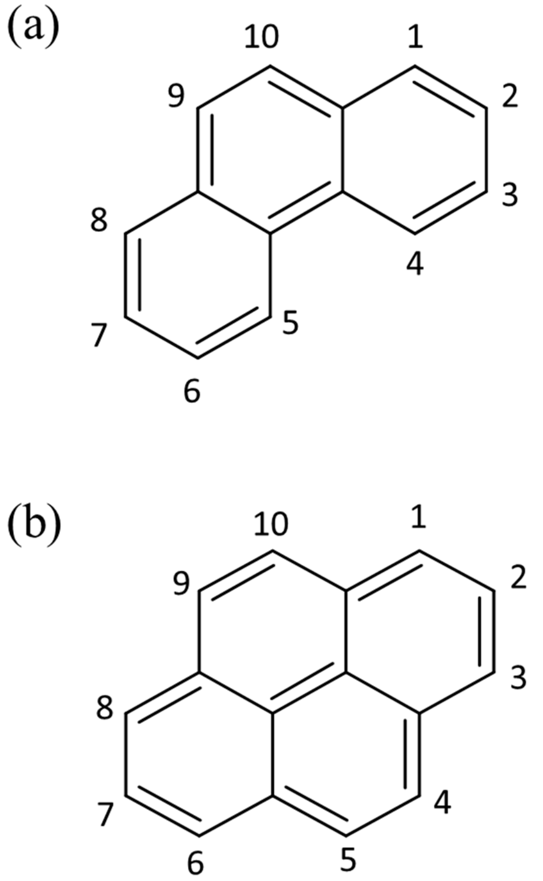

Figure S8. The chemical structures of (a) phenanthrene and (b) pyrene with their carbon numbering.
